# Extrinsic allospecific signals of hematopoietic origin dictate iNKT cell lineage-fate decisions during development

**DOI:** 10.1038/srep28837

**Published:** 2016-06-29

**Authors:** Beverly S. I. Strong, Tess J. Newkold, Amanda E. Lee, Lucas E. Turner, Amir M. Alhajjat, Jonathan W. Heusel, Aimen F. Shaaban

**Affiliations:** 1 Department of Surgery, Cincinnati Children’s Hospital Medical Center and the University of Cincinnati College of Medicine, Cincinnati, Ohio 45229, USA; 2 Department of Surgery, University of Iowa Carver College of Medicine, Iowa City, Iowa 52242, USA; 3 Departments of Pathology & Immunology and Genetics, Washington University School of Medicine, St. Louis, Missouri, 63110, USA

## Abstract

Invariant NKT (iNKT) cells are critical to the maintenance of tolerance toward alloantigens encountered during postnatal life pointing to the existence of a process for self-education. However, the impact of developmentally encountered alloantigens in shaping the phenotype and function of iNKT cells has not been described. To better understand this process, the current report examined naïve iNKT cells as they matured in an allogeneic environment. Following the prenatal transfer of fetal hematopoietic cells between age-matched allogeneic murine fetuses, cell-extrinsic signals appeared to dictate allospecific patterns of Ly49 receptor expression and lineage diversity in developing iNKT cells. Regulation for this process arose from cells of hematopoietic origin requiring only rare exposure to facilitate broad changes in developing iNKT cells. These findings highlight surprisingly asymmetric allospecific alterations in iNKT cells as they develop and mature in an allogeneic environment and establish a new paradigm for study of the self-education of iNKT cells.

Invariant natural killer T cells (iNKT) have been shown to mediate immune responsiveness or tolerance in alternate settings^1^. The recognition and activation pathways of iNKT cells are evolutionarily conserved among various species and offer a bridge between innate and adaptive immunity. Specifically, in hematopoietic cellular transplantation in mice and humans, iNKT cells appear to play a critical role suppressing graft-versus-host disease (GVHD) through production of T_H_2 cytokines and providing support for regulatory T cells or tolerogenic dendritic cells^2^,^3^,^4^,^5^,^6^,^7^. While the tolerogenic role of iNKT cells following transplantation is apparent, a fundamental delineation of the regulatory receptor-ligand interactions leading to the self-education of developing iNKT cells remains elusive. The information gap widens when considering the complexities of iNKT cell maturation and function in the allogeneic environment.

Proposed pathways for self-recognition or alloreactivity of iNKT cells in mice include diversity of lipid-antigen recognition through the invariant TCR, inhibitory Ly49 (iLy49) interaction with class I ligands, and variation in iNKT lineage repertoire. iNKT cells express a restricted set of TCRs with specificity for lipid antigens presented by the non-classical MHC molecule CD1d^8^,^9^,^10^. Glycolipid antigens can be derived from gram-negative bacteria that synthesize α-anomeric glycolipids such as α-galactosylceramide (α-GalCer) which is derived from Sphingomonas capsulata, or endogenous glycolipid self-antigens like isoglobotrihexosylceramide^11^,^12^,^13^,^14^. The nature of the functional response by iNKT cells (pro-inflammatory or immunosuppressive) is dictated by the binding kinetics of the individual glycolipid antigens to CD1d^12^.

Strain-specific MHC class I alleles provide a pathway for allorecognition by Ly49 receptors expressed by iNKT cells. Unlike NK cells, iNKT cells only express inhibitory Ly49 receptors and lack activating receptor expression. Indeed, forced expression of the Ly49D receptor by immature thymocytes inhibits CD1d-restricted T cell development in a ligand-dependent manner indicating that activating Ly49 receptor signaling is incompatible with iNKT cell development^15^,^16^. Co-expression of the Ly49A inhibitory receptors that shares specificity with Ly49D for H-2D^d^ MHC class I antigen rescues iNKT cell development in the same model suggesting functionality of inhibitory Ly49 signaling in iNKT development^15^,^16^. Further support for functional importance of Ly49 receptors on iNKT cells is derived from observations of reduced activation exhibited by receptor-bearing iNKT cells in the presence of cognate MHC ligand^17^,^18^. The biological significance of this consistent observation remains incompletely understood. Lastly, although human iNKT cells display alloreactivity mediated by homologous killer immunoglobulin-like (KIR) receptors, direct alloreactivity of murine iNKT cells has not been demonstrated^19^,^20^.

Self-tolerance through differential responsiveness in various strains of mice may also arise as iNKT cells mature into distinct lineages during development. Mature iNKT cells can be grouped into 3 dominant distinct lineages (NKT1, NKT2, and NKT17) according to their expression of the transcription factors PLZF and T-bet. NKT1 cells (PLZF-low, Tbet-high) primarily produce IFN-γ. NKT2 cells (PLZF-high, Tbet-low) produce IL-4, while NKT17 cells (PLZF-low Tbet-low) make IL-17^21^,^22^,^23^,^24^,^25^. The lineage diversity between inbred mouse strains differs dramatically suggesting that these patterns result from genetic differences between the strains^21^. However, a role for environmentally-derived signals in guiding fate decisions made by developing iNKT cells has not been well-studied.

The current report examined the allospecific education and functional maturation of iNKT cells using a mouse model of in utero hematopoietic cell transplantation (IUHCT) that involved prenatal transfer of hematopoietic cells between age-matched fetuses before the onset of thymic TCR rearrangement facilitating analysis of the ensuing patterns of iLy49 receptor co-expression, glycolipid responsiveness and lineage-diversity of iNKT cells. The relative strength of this approach emerges in the comparison between responsive and irrelevant iNKT cells during their parallel development within the same chimeric animal. The findings of this report reveal that cell-extrinsic signals dictate patterns of Ly49 receptor expression and lineage diversity in developing iNKT cells.

## Results

### The level of allospecific Ly49 receptor expression is altered on host iNKT cells in prenatal chimeras

**Figure 1:**
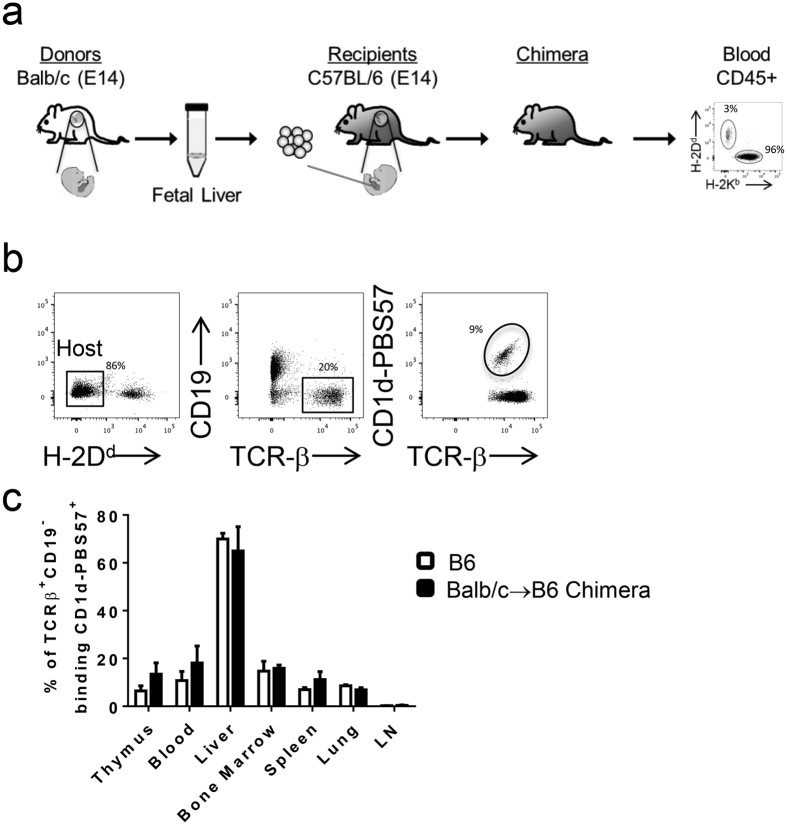
Stable frequency of iNKT cells in prenatal Balb/c --> B6 prenatal hematopoietic chimeras. (**a**) Schematic of transplantation model where E14 Balb/c fetal liver cells were transplanted into age-matched B6 fetuses via an intrahepatic route. (**b**) PB chimerism was assessed by measuring the frequency of hematopoietic cells expressing MHC I (H-2K^d^ or H-2D^d^) starting at 3 weeks of age. Host iNKT were identified as H2-K^d−^ CD19^−^ TCRβ^+^ CD1d-PBS157 tetramer^+^. (**c**) Multi-organ analysis demonstrating frequencies of iNKT between Balb → B6 prenatal chimeras and age-matched B6 control mice (percent of total TCRβ+ events that are CD1d-PBS57+). Data shown is representative of three 14 week mice per group.

This study employed an established Balb/c → B6 model of allogeneic IUHCT to evaluate the education of iNKT cells and their role in prenatal tolerance (Fig. 1a). In this model, E14 fetal liver cells were isolated from Balb/c donor fetuses and transplanted into age-matched B6 fetuses. Animals were allowed to progress toward delivery and PB chimerism was assessed after weaning. Host and donor populations were identified by expression of strain-specific MHC class I. Chimerism levels were approximately 2–15% in most tissues tested with the exception of thymic chimerism which was naturally low (Supplemental Fig. 1.) iNKT cells were identified by TCR-β expression and binding to CD1d-tetramers loaded with PBS57 glycolipid (Fig. 1b). Host iNKT cells were detected in chimeras and control animals at similar frequencies in all organs analyzed (Fig. 1c).

**Figure 2:**
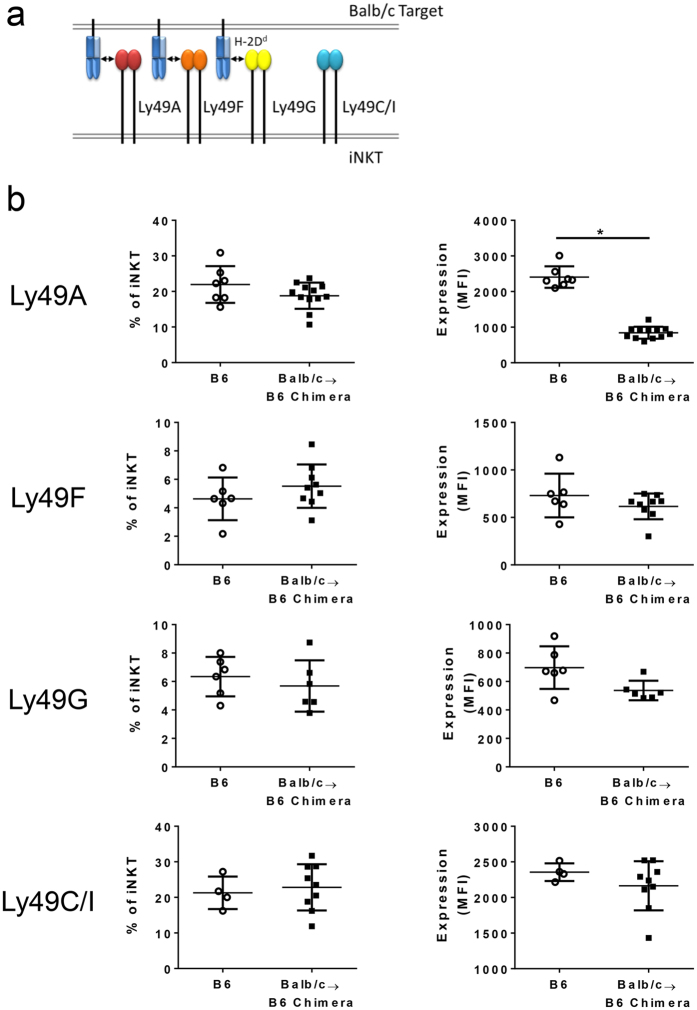
Reduced expression of allospecific Ly49 receptors on iNKT cells in PB of prenatal chimeras. (**a**) Schematic of Ly49 reactivity to donor Balb/c antigens. The Ly49A, Ly49F, and Ly49G receptors expressed by B6 iNKT cells are reactive toward *H2*^*d*^ MHC class I ligands expressed by the Balb/c hematopoietic cells. The Ly49C/I receptors are not reactive toward *H2*^*d*^ ligands. (**b**) Frequency of receptor-positive cells and level of expression on individual iNKT cells (mean fluorescence intensity (MFI)) in PB is compared between Balb/c → B6 prenatal chimeras and age-matched B6 control mice. Each data point represents one 12–16 week mouse. All data is gated on live CD19^−^ TCR-β^+^ CD1d-PBS57^+^ events.

In this Balb/c → B6 model of IUHCT Ly49A, F, and G on host iNKT cells bind specifically to donor-derived H-2^d^ MHC class I while Ly49C/I lacks a specific ligand on the donor cells (Fig. 2a). Expression of allospecific Ly49 receptors by PB host iNKT cells was compared between chimeras and controls. While there was no significant change in the frequency of iNKT cells expressing an allospecific Ly49 receptor, there was a significant reduction in the level of Ly49A expression by individual iNKT cells developing in the chimeric environment (Fig. 2b) No changes in Ly49F or Ly49G expression were observed between the groups. Furthermore, no changes occurred in the non-specific Ly49C/I receptor frequency or expression.

**Figure 3:**
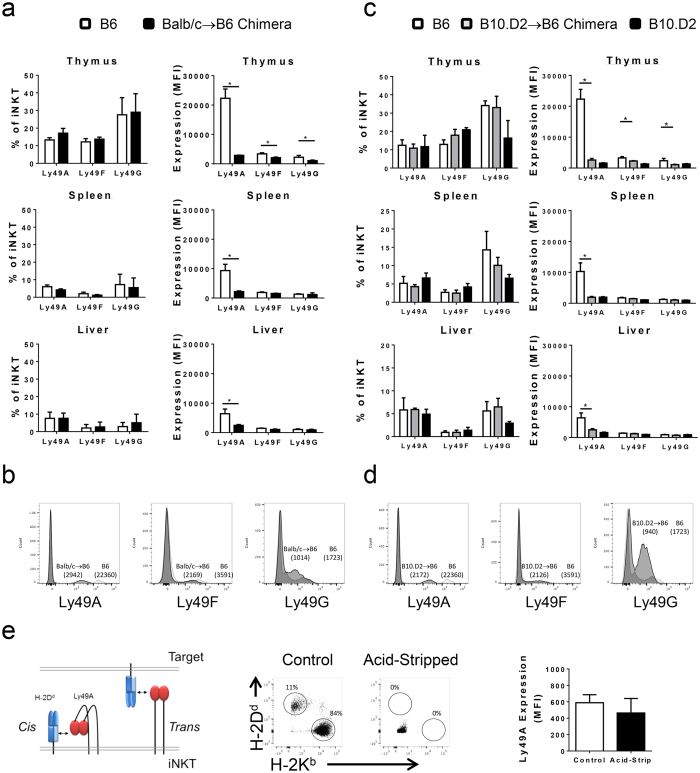
Allospecific Ly49 receptors are reduced developmentally in the thymus with continued Ly49A downregulation in the periphery. (**a**) Frequency of iNKT cells expressing allospecific Ly49A, F, or G receptors in thymus, spleen, or liver from Balb/c → B6 prenatal chimeras. Expression of Ly49A, F, or G receptors (MFI) on individual iNKT cells from the thymus of prenatal chimeras and controls. (**b**) Representative histograms demonstrating the MFI of Ly49 expression on thymic iNKT cells in B6 control and Balb/c → B6 chimeras. Numbers indicate MFI. (**c**) The frequency and MFI of Ly49A+ iNKT cells in B6 controls, B10.D2 → B6 chimeras, and B10.D2 controls. (**d**) Representative histograms demonstrating the MFI of Ly49 expression on thymic iNKT cells in B6 control and B10.D2 → B6 chimeras. Numbers indicate MFI. (**e**) Schematic of Ly49A capacity to bind in *trans* to MHC class I on target cells and in *cis* to MHC class I expressed on the cell surface of the same iNKT cell. Acid stripping was used to dissociate MHC class I resulting in the elimination of potential cis-interactions of Ly49A with MHC class I. Expression (MFI) of the Ly49A receptor on splenic iNKT in prenatal chimeras after control versus acid-stripping conditions. All data is representative of three mice per group. All data in (**a–d**) is gated on CD19^−^ TCRβ^+^ CD1dPBS57^+^ iNKT; data in (**e**) is gated on CD19-TCRβ+ NK1.1+ events.

To track the developmental origins of these changes, thymic analysis was performed to assess the earliest expression of Ly49 receptors on iNKT cells before migration to peripheral organs^26^. Thymic iNKT cells in prenatal chimeras displayed minimal changes in the frequency of allospecific Ly49F+ cells when compared to controls (Fig. 3a). However, the expression of all allospecific Ly49 receptors was significantly decreased on individual thymic iNKT cells in chimeric mice when compared to controls (Fig. 3a,b). This pattern contrasted sharply with the relative homogeneity of receptor expression seen in the spleen and liver for Ly49F and G suggesting that alteration in the expression of these alloreactive receptors was regulated during thymic development. To confirm that developmental regulation of Ly49A was due to the specific presence of H-2^d^ MHC class I, iNKT cells were analyzed in B10.D2 → B6 chimeras (Fig. 3c,d). B10.D2 mice express H-2^d^ MHC class I without the presence of other alloantigens present in Balb/c mice. Host iNKT cells in B10.D2 → B6 chimeras did not exhibit changes in the frequency of Ly49A, F, or G expression. Similar to the Balb/c → B6 chimeras, there was a significant decrease in the MFI of Ly49A, F, and G expression on host thymic iNKT cells compared to B6 controls. In the periphery, there was a selective reduction in Ly49A in spleen and liver. Collectively, these data indicate that changes in alloreactive Ly49 receptor expression are due to the presence of H-2^d^ MHC class I and not the result of minor antigen disparities.

Ly49A contains a hinge region permitting both trans-binding to MHC class I on target cells or cis-binding to self-ligands expressed on the same cell (Fig. 3e). While host cells do not endogenously express the H-2^d^ ligand, trogocytosis may result in low levels of donor-derived MHC class I ligand on host NKT cells^27^. To determine if cis-interaction following MHC-transfer contributed to the apparent downregulation of Ly49A in the periphery, the chimeric cells were subjected to the same analysis after acid-stripping. Brief acid-stripping dissociates MHC class I molecules unmasking any Ly49A receptor that may be sequestered by cis-binding^28^. Since acid-treatment also interferes with tetramer binding and consequently the exclusive identification of iNKT cells, a bulk analysis of the NK1.1+ TCRβ+ parent population was performed. As shown in Fig. 3e, there was no change in the expression of Ly49A following acid-stripping confirming the reduced expression on NKT cells in chimeras. Together, these data indicate that Ly49A receptor levels in iNKT cells are developmentally altered in response to prenatally introduced cognate MHC class I antigen.

### The level of expression of allospecific Ly49A is reduced on donor iNKT cells in prenatal chimeras

**Figure 4:**
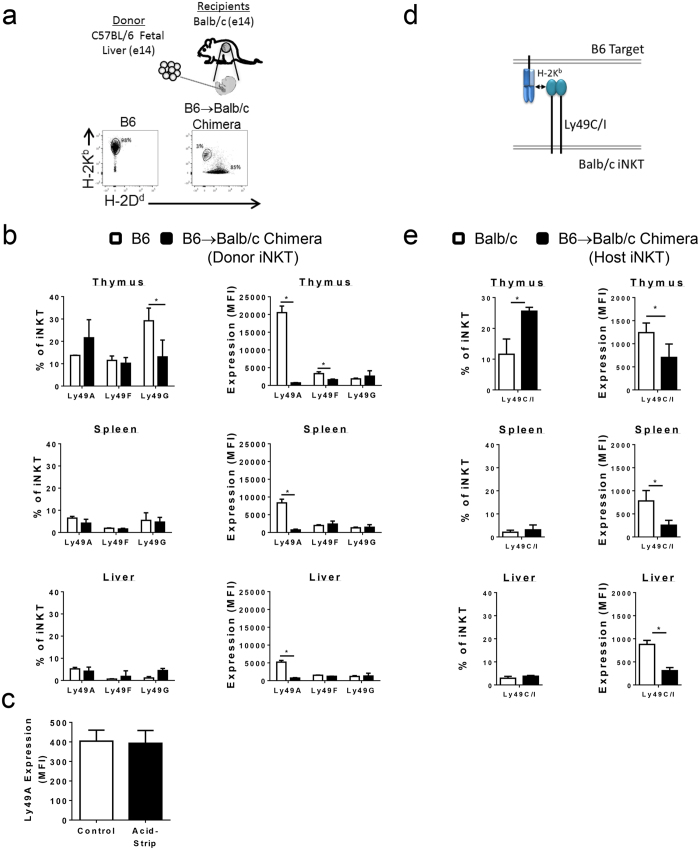
Expression of allospecific Ly49A receptor is reduced on donor iNKT in B6 → Balb/c chimeras while allospecific Ly49C/I receptor is reduced on host iNKT in B6 → Balb/c chimeras. (**a**) Schematic of transplantation model of B6 E14 fetal liver into age-matched Balb/c fetal recipients. Representative dot plots of gating for donor cells in B6 → Balb/c chimeras. (**b**) Frequency of Ly49A+, F+, and or G+ cells amongst all donor iNKT cells in prenatal chimeras compared to age-matched B6 control mice. The expression of Ly49A, F, or G receptors on individual donor iNKT cells in thymus, spleen and liver of B6 → Balb/c chimeras. (**c**) Ly49A expression by donor splenic iNKT cells from chimeric mice after control versus acid-stripping conditions as described in Fig. 3. (**d**) Schematic of Ly49 receptor expression by Balb/c iNKT cells and reactivity with B6 MHC class I alleles. (**e**) Frequency of Balb/c iNKT cells expressing allospecific Ly49A, G, or C/I receptors in thymus, spleen, or liver from Balb/c → B6 prenatal chimeras and Balb/c controls. Expression of Ly49A, G, or C/I receptors (MFI) on individual Balb/c iNKT cells from the thymus of B6 → Balb/c chimeras and Balb/c controls. N.D. indicates not detected consistent with the absence of the Ly49F gene in the Balb/c genome. All data is representative of three to six mice per group. All data in (**b,c**) is gated on B6 CD19^−^ TCRβ^+^ CD1dPBS57^+^ iNKT cells while all data in (**e**) is gated on Balb/c CD19^−^TCRβ^+^ CD1dPBS57^+^ iNKT cells.

To determine the effect of much higher levels of cognate class I ligand, allospecific Ly49A receptor expression was measured in donor iNKT cells from reverse B6 → Balb/c chimeras where *H2*^*d*^ antigens are ubiquitous in the recipient environment (Fig. 4a). Donor B6 iNKT cells expressing Ly49A, F, and G had similar frequencies compared to B6 controls with the exception of a decrease in Ly49G in the thymus. No significant changes were seen in the remaining organs tested (Fig. 4b). Similar to what was seen with host iNKT cells, the level of Ly49A expression exhibited by individual donor iNKT cells was decreased in the thymus, spleen, and liver (Fig. 4b). Ly49F MFI was also reduced selectively in the thymus. Similarly, acid-stripping did not result in a change in Ly49A MFI indicating that reduced staining was likely due to decreased receptor expression and not cis-binding (Fig. 4c). Together these data indicate that allospecific inhibitory Ly49 expression by donor iNKT cells is reduced similarly in the presence of low levels of ligating MHC class I in the Balb/c → B6 chimeras and high levels in the B6 → Balb/c reverse chimeras.

### Expression of allospecific receptors by Balb/c iNKT cells

Balb/c mice express Ly49C/I, a receptor with specificity for B6 H-2K^b^ MHC class I (Fig. 4d). The expression of this allospecific receptor was compared between host Balb/c iNKT cells in B6 → Balb/c chimeras and Balb/c controls to evaluate the impact on Ly49C/I expression following developmental exposure to cognate ligand. Selectively in the thymus, there was an elevation in the frequency of Ly49C/I expression in chimeras that was not maintained in the periphery (Fig. 4e). However, the expression of allospecific Ly49C/I receptor was consistently downregulated on individual thymic and peripheral iNKT cells in chimeric mice when compared to Balb/c controls. These data further support the principle that developmental exposure to ligand leads to changes in iLy49 receptor expression by iNKT cells.

### Heterogeneity of Ly49 receptor expression by iNKT cells

**Figure 5:**
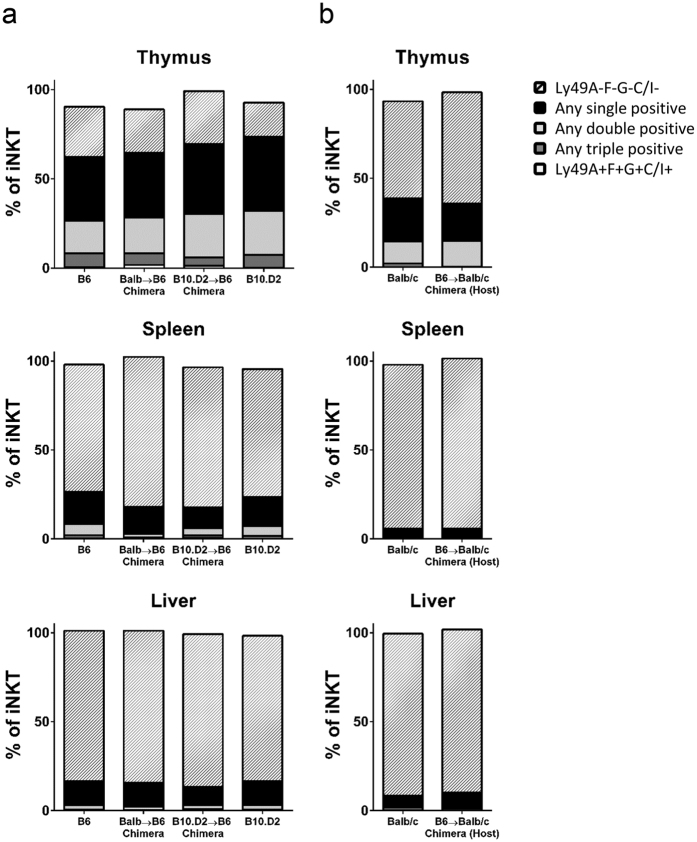
Heterogeneity of Ly49 receptor expression by iNKT cells in prenatal chimeras. Control (B6, B10.D2, and Balb/c) and chimeric (Balb/c → B6, B10.D2 → B6, and B6 → Balb/c) mice were analyzed for expression of individual Ly49 receptors. Percentage of iNKT cells expressing none (A-F-G-C/I-), any single Ly49 receptor (i.e. Ly49A+ F− G− C/I−), any two receptors (i.e. Ly49A+ F+ G− C/I−), any three Ly49 receptors (i.e. Ly49A+ F+ G+ C/I−), or all four receptors (Ly49A+ F+ G+ C/I+) in thymus, spleen, and liver. (**a**) Data in chimeras is gated on the host B6 cells. (**b**) Data in chimeras is gated on host Balb/c cells. All data is gated on iNKT cells and is representative of at least three mice.

The heterogeneity of Ly49A, F, G, and C/I receptor expression was evaluated on iNKT cells during development in the thymus and in the periphery. In the thymus, the majority of B6 iNKT cells expressed at least one Ly49 receptor in B6 controls, Balb/c → B6 and B10.D2 →  B6 chimeras (Fig. 5a). Of the receptor-bearing thymic iNKT cells, most expressed only a single Ly49 receptor with a lower frequency of cells expressing two, three, or all four receptors. In striking contrast, B6 iNKT cells in the periphery predominantly lacked expression of any of the Ly49 receptors. This pattern of expression was also observed in B10.D2 control mice. Balb/c iNKT cells were also compared between controls and B6 → Balb/c chimeras (Fig. 5b). Balb/c mice lack the gene for Ly49F, so analysis was limited to expression of Ly49A, G, and C/I. Similar to B6 iNKT cells, approximately 50% of thymic iNKT cells expressed at least one Ly49 receptor with most receptor bearing cells expressing only a single receptor. In the periphery, the frequency of receptor-bearing iNKT cells fell dramatically. There were no changes in the overall distribution of receptor heterogeneity between Balb/c controls and chimeras. The consistency of the receptor heterogeneity between chimeras and controls suggests that this is an intrinsic feature of the iNKT cell development and not due to the environmental presence of ligating MHC class I.

### Binding of allospecific Ly49 receptors during development does not alter the functional response of iNKT cells

**Figure 6:**
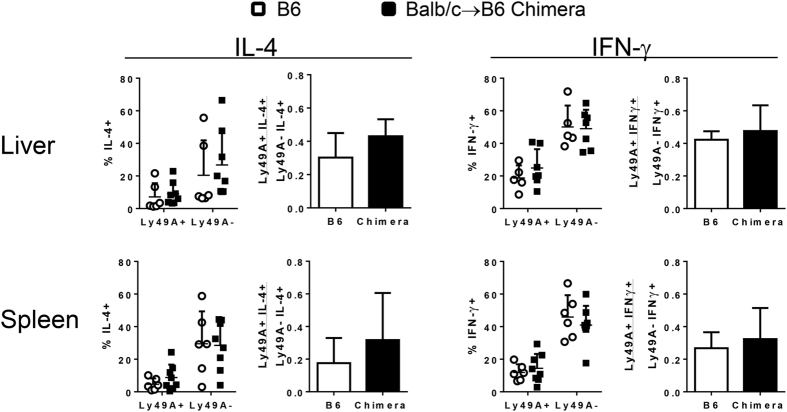
Functional responsiveness of NKT in prenatal chimeras. B6 control and Balb/c → B6 chimeras were injected intravenously with KRN7000 and NKT cells were analyzed two hours later for intracellular cytokine production. NKT cells were identified by co-expression of TCR-β and NK1.1 rather than CD1d-PBS57 tetramer due to down-regulation of TCR following stimulation. Absolute and relative frequency of IFN-γ+ and IL-4+ NKT cells are shown in graphs. Relative frequency of cytokine expression was calculated for donor reactive versus donor irrelevant phenotypes (Ly49A+% Cytokine+/Ly49A−% Cytokine+). Each data point represents one animal.

Ly49 expressing iNKT cells have been shown to be hyporesponsive both in terms of proliferation and cytokine production^17^,^18^. However, the potential of developmental exposure to high-affinity ligand on iNKT responsiveness has not been assessed. To determine if the observed phenotypic reduction of Ly49A on host iNKT in prenatal chimeras carries functional significance, iNKT cells were stimulated *in vivo* with synthetic α-GalCer (KRN7000). The cytokine response was gauged as the total cytokine production and also as a ratio of cytokine produced by allospecific Ly49A+/irrelevant Ly49A- host B6 NKT cells. Using either of these measures, the production of IFN-γ and IL-4 by liver and spleen Ly49A+ or Ly49A- NKT cells was found to be similar between chimeras and naïve controls (Fig. 6). The frequency of IFN-γ+ or IL-4+ cells amongst Ly49A+ NKT cells was nearly equal while a heterogeneity of responsiveness was exhibited by the Ly49A- NKT cells (Fig. 6). To exclude the possibility that differences between the groups were masked by the analysis of only a single alloreactive receptor, the impact of co-expression of additional alloreactive receptors was assessed. Similar results were seen when all alloreactive NKT cells were analyzed—with alloreactive cells identified by expression of any combination of Ly49A, F, or G receptors (AFG+) and donor-irrelevant cells identified by the absence of all of these receptors (AFG-; Supplementary Fig. 2). Stimulation with KRN7000 resulted in the inability to identify iNKT cells with the CD1d tetramer, leading to the identification of total NKT cells by NK1.1 staining. In order to more directly assess the activation of iNKT cells, a less potent analog of αGalCer (OCH) was used to stimulate iNKT cells *in vivo*^29^. Following OCH stimulation, it was possible to gate on tetramer positive iNKT cells (Supplemental Fig. 3a). Consistent with the results using KRN7000 stimulation, the production of IFN-γ and IL-4 by liver and spleen Ly49AFG+ or Ly49AFG- iNKT cells was found to be similar between chimeras and controls (Supplemental Fig. 3b). Together these data indicate that iNKT cells in prenatal chimeras retain normal responsiveness to TCR stimulation despite altered levels of alloreactive inhibitory Ly49 receptor expression.

### Cell-extrinsic allospecific signals shape iNKT cell lineage-fate decisions in chimeric mice

**Figure 7:**
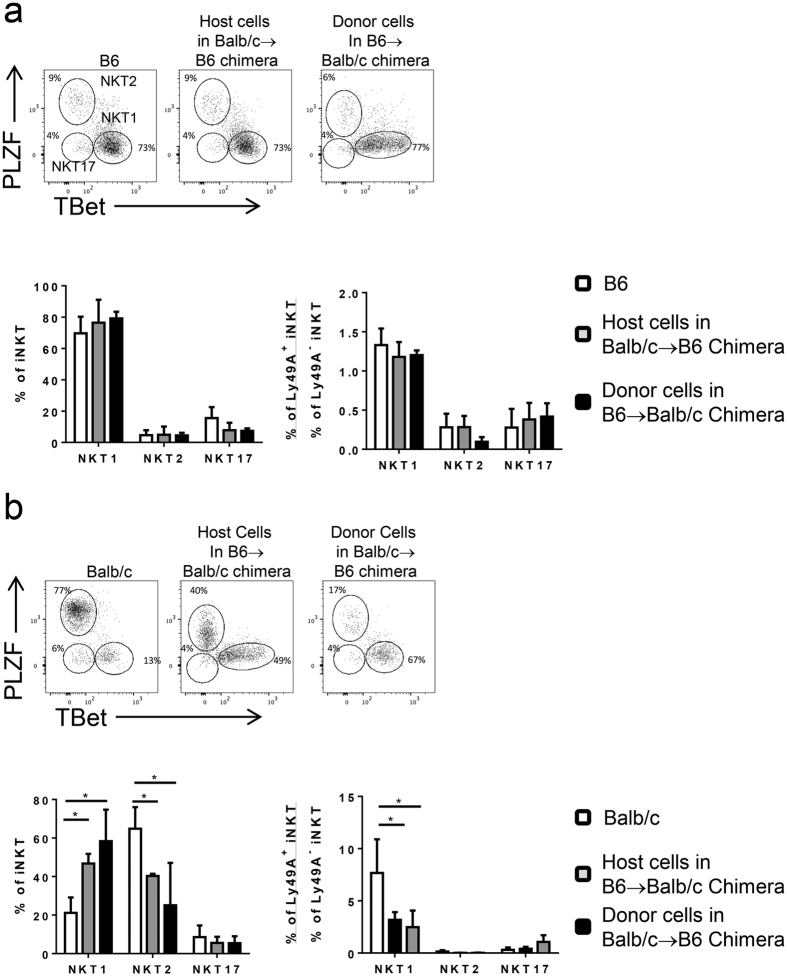
iNKT lineage distribution is asymmetrically altered on Balb/c but not B6 cells in prenatal chimeras. (**a**) Comparison of thymic iNKT cell lineage distribution of B6 iNKT cells from control mice, Balb/c → B6 chimeras (host cells), B6 → Balb/c prenatal chimeras (donor cells) as identified by intranuclear PLZF and T-bet staining. Data is summarized as absolute frequency and ratio of donor-reactive versus donor-irrelevant (Ly49A+/Ly49A−) iNKT cells in chimeras and controls. (**b**) Thymic iNKT cell lineage distribution of Balb/c iNKT cells from control mice, B6 → Balb/c (host cells), and Balb/c → B6 (donor cells) chimeras. All data is gated on CD19^−^ TCRβ^+^ CD1dPBS57^+^ iNKT events from 7–10 weeks of age and is representative of 4–6 mice per group.

B6 and Balb/c mice represent extreme ends of the spectrum of iNKT lineage diversity with B6 being NKT1-dominant and Balb/c NKT2-dominant mirroring these mouse strains general immune skewing toward T_H_1 versus T_H_2 responses, respectively^30^,^31^. Therefore, thymic iNKT cell lineage diversity was compared between chimeric and control mice to determine if the lineage commitment of immature iNKT cells was intrinsically determined or extrinsically regulated by the allospecific environment during development. Following prenatal transplantation, B6 thymic iNKT cells developing as either donor or host cells in allogeneic chimeric mice retained the same pattern of lineage diversity as naïve B6 controls (Fig. 7a). Additionally, a comparison of the lineage distribution between donor-reactive Ly49A+ and donor—irrelevant Ly49A- iNKT cells in the same chimeric mice revealed no differences from controls (Fig. 7a). Conversely, Balb/c iNKT cells developing in allogeneic prenatal chimeras exhibited a shift toward a NKT1 lineage predominance characteristically seen in B6 mice and away from a NKT2 lineage bias seen in naïve Balb/c controls (Fig. 7b). These alterations occurred with Balb/c cells in both the donor and the host situation. Furthermore, NKT1 cells were predominantly Ly49A+ in naïve Balb/c mice, but this correlation was reduced in chimeric animals more closely approximating the ratio seen in B6 mice. Collectively, these findings demonstrate the potential for cell-extrinsic signals in guiding iNKT cell lineage fate in an asymmetric fashion.

### iNKT cells are not required for engraftment or tolerance to prenatal transplantation

**Figure 8:**
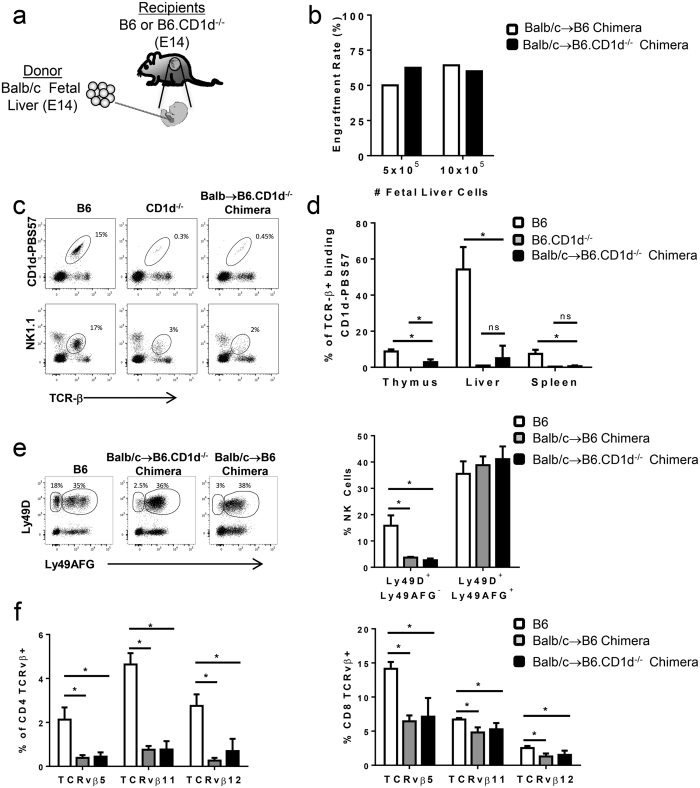
iNKT cells are not required for tolerance to prenatal transplantation. (**a**) Schematic of transplantation model of Balb/c E14 fetal liver hematopoietic cells into age-matched B6.CD1d^−/−^ fetal recipients. (**b**) Engraftment rate at 12 weeks in Balb/c → B6.CD1d^−/−^ and Balb/c → B6 chimeras. (**c**) Representative dot plots demonstrating reduced frequency of iNKT cells in liver of B6.CD1d^−/−^ mice and Balb/c → B6.CD1d^−/−^ chimeras. (**d**) Frequency of iNKT cells in thymus, liver, and spleen of Balb/c → B6.CD1d^−/−^ chimeras relative to B6 mice and B6.CD1d^−/−^ mice. (**e**) Hostile (Ly49D+ AFG−) and tolerant (Ly49D+ AFG+) NK cell phenotype in the PB of B6.CD1d^−/−^ chimeras relative to B6 control mice and B6 chimeras. (**f**) Deletion of allospecific CD4 and CD8 T cells in B6.CD1d^−/−^ and B6 chimeras relative to B6 controls. All data is representative of at least three mice per group.

The TCR expressed by iNKT cells recognizes glycolipids in the context of CD1d and is essential for iNKT cell development^32^. To determine the role of iNKT cells in tolerance to prenatal allogeneic transplantation, Balb/c → B6.CD1d^−/−^ (B6.CD1d^−/−^ chimeras) transplants were generated (Fig. 8a). No difference in engraftment rates or survival were observed between CD1d^−/−^ and control chimeras at two different cell doses (Fig. 8b). When B6.CD1d^−/−^ chimeras were analyzed, a >95% reduction in iNKT cell frequency was observed in the thymus, liver, and spleen (Fig. 8c,d). There was no significant difference in the minor frequency of iNKT cells present in the livers and spleens Balb/c → B6.CD1d^−/−^ chimeras compared to B6.CD1d^−/−^ controls. However, a small population of iNKT cells was present in the thymus which could have been generated in response to the presence of CD1d antigen on donor Balb/c cells.

Next, the requirement for iNKT cell support for allospecific NK and T cell education was compared between B6.CD1d^−/−^ chimeras and B6 chimera controls^33^,^34^. No differences were observed in the pattern of NK or T cell education between control and B6.CD1d^−/−^ chimeras. Specifically, in both types of chimeric recipients, there was a dramatic reduction in the frequency of NK cells expressing the allospecific Ly49 activating receptor without co-expressing an allospecific inhibitory receptor (Ly49D+ Ly49AFG-, Fig. 8e). These NK cells are phenotypically “hostile” towards the Balb/c transplanted cells and are detrimental to transplant tolerance^33^,^35^. The frequency of phenotypically “friendly” NK cells that co-express both an allospecific activating and inhibitory receptor (Ly49D+ Ly49AFG+) was also the similar between B6.CD1d^−/−^ chimeras and B6 chimera controls (Fig. 8e). Furthermore, T cell populations expressing TCRvβ5, 11, and 12 react with endogenous mammary tumor virus-derived superantigens bound to I-E family MHC class II molecules on donor Balb/c cells resulting in the deletion of these populations in Balb/c → B6 chimeras^34^,^36^,^37^,^38^. Both wild-type and B6.CD1d^−/−^ chimeras displayed deletion of allospecific TCRvβ5, 11, and 12 T cells (Fig. 8f) with no significant difference between B6 and B6.CD1d^−/−^ chimeras. Together these data indicate that iNKT cells are likely dispensable for tolerance to prenatally encountered alloantigens.

## Discussion

While developing in the prenatal chimeric environment, iNKT cells must simultaneously acquire tolerance to both the donor and host antigens while retaining full responsiveness to immunostimulatory signals. Given the invariant nature of TCR usage, the tolerogenic changes occurring in iNKT cells are likely limited to variations in inhibitory Ly49 receptor expression and iNKT cell lineage diversity. In this study, developmental exposure of B6 (*H2*^*b*^) iNKT cells to allogeneic Balb/c (*H2*^*d*^) cells led to alterations in Ly49A receptor expression illustrating the educational impact of ligand recognition by inhibitory Ly49 receptors. Decreased levels of allospecific Ly49F and G were also detected in the thymus but not in the periphery possibly due to the relatively lower affinity of these receptors for the donor ligands^39^. Despite these changes, iNKT cells from B6 chimeric mice displayed no alteration in cytokine responses following TCR stimulation. Significantly, these findings highlight the different role of inhibitory Ly49 receptors on NK versus iNKT cells. Lastly, although the developmental ligation of inhibitory Ly49 receptors on NK cells has been shown to enhance functional responsiveness (licensing)^40^, the ligation of inhibitory Ly49 receptors on iNKT cells during development did not alter the response to TCR stimulation. It remains possible that ligation of Ly49 receptors regulates responsiveness of iNKT cells to other activating signals.

Inhibitory Ly49 expression by NKT cells appears to be developmentally regulated with higher expression exhibited in the thymus than in the periphery^17^,^18^,^26^,^41^. Limited information suggests that the expression of cognate MHC class I ligand leads to downregulation of self-specific Ly49 receptor expression on individual iNKT cells in the thymus and the periphery^17^,^26^. The findings of this study support this contention as the level of Ly49A expression was downregulated on B6 iNKT cells while Ly49C/I was downregulated on Balb/c iNKT cells in the thymus and periphery of prenatal allogeneic chimeras. The lack of change in the frequency of receptor-positive iNKT cells in our system may be due to the relatively low levels of the Balb/c ligands in Balb/c → B6 chimeras. Furthermore, these data demonstrate that ligation of Ly49A primarily via *trans* interactions on the surface of iNKT cells is sufficient for receptor downregulation. Previous studies were performed in systems where Ly49 receptors interacted with MHC class I ligand in both *trans* and *cis*. In the chimeric model, donor Balb/c cells exclusively express *H2*^d^ with only very low levels of MHC transferred via trogocytosis to B6 iNKT cells^27^. Lastly, these data also demonstrate that a similar downregulation of Ly49A expression occurred when the encounter with class I ligand was rare (Balb/c → B6 chimeras) or when it was ubiquitous (B6 → Balb/c chimeras).

The current study provides the intriguing finding that iNKT cell lineage diversity was asymmetrically affected by the chimeric environment. Specifically, B6 iNKT lineage diversity was intrinsically determined and unaffected by exposure to allogeneic Balb/c cells during development while the Balb/c iNKT cell lineage profile appeared to be extrinsically determined and dominantly skewed toward an NKT1 lineage following maturation in a B6 host. Assuming a singular system exists, the extrinsic regulatory control for NKT1 lineage skewing afforded by the B6 environment must be of hematopoietic cell origin as similar changes were seen in Balb/c iNKT cells in either the donor or host situation. The exposure to even small numbers of B6 cells appeared to override the contribution of Ly49A to developmental decisions made by Balb/c iNKT cells illustrating the efficiency of this mechanism.

Although a myriad of cell-intrinsic signals are known to regulate lineage diversity in iNKT cells, little is known regarding the potential for extrinsic signals to direct iNKT lineage-fate decisions^21^,^42^,^43^,^44^. One potential extrinsic pathway relates to the demonstration that exogenous IL-15, vitamin D, and retinoic acid induce the upregulation of let-7 microRNA (miRNA) and that a loss of let-7 expression in B6 mice results in a decrease in the frequency of NKT1 cells and a corresponding increase in NKT2 and NKT17 cells^45^. In an earlier study, loss of IL-15 expression by thymic stroma led to alterations in the ratio of T-bet to Gata-3 expressing thymic iNKT cells potentially influencing lineage diversity^46^. The potential for tissue-specific stroma to guide lineage diversity is further supported from the observation that peripheral localization of iNKT cells in both B6 and Balb mice is skewed with NKT1 cells almost exclusively populating the liver while Peyer’s patches have a mixed presence of NKT1, NKT2, and NKT17 cells^47^. However, it is unlikely that a tissue-specific mechanism accounts for the extrinsic influence provided by the transplanted B6 hematopoietic cells in the B6 → Balb/chimeras. Lastly, allelic variations in SLAM family members have been shown to alter iNKT cell lineage diversity in NOD and 129 mice, but the impact of SLAM haplotypes on B6 versus Balb NKT cell development has not been thoroughly evaluated^48^,^49^,^50^. Future studies are needed to determine if B6 and Balb/c mice display inherent differences in these cell-extrinsic pathways that might account for the B6 dominant skewing of Balb/c iNKT cells.

It has been proposed that unique TCR signals dictate lineage fate decisions by iNKT cells resulting in marked differences in the distribution between inbred mouse strains^21^. While iNKT cells respond to α-GalCer, share an invariant TCRα chain, and possess limited heterogeneity in TCRβ chain usage, there remains significant diversity in the complementarity determining region (CDR) 3β regions, providing the potential for recognition of distinct glycolipids and/or alterations in the affinity of TCR binding^10^,^51^,^52^. NKT2 cells in both B6 and Balb mice express higher levels of Nur77 and represent a greater percentage of TCRvβ2 and TCRvβ7 expressing iNKT cells suggesting additional pathways through which unique TCR signals may influence lineage decisions by iNKT cells^21^.

Lastly, although previous studies have demonstrated that iNKT cells are required for tolerance in postnatal bone marrow transplantation^2^,^3^,^4^,^5^, the present findings strongly suggests that iNKT cells are not required for tolerance to prenatally encountered antigens as there were no changes in engraftment prevalence, NK cell tolerance, or T cell tolerance in the iNKT-deficient B6.CD1d^−/−^ prenatal chimeras. One possible explanation for these disparate findings is that the technical approach to prenatal transplantation is intrinsically less destructive to the host environment than postnatal transplantation. Whole-body irradiation, a critical component of most bone marrow transplantation protocols, itself induces inflammation^53^. Since inflammatory cytokines are capable of directly activating iNKT cells, a greater role for iNKT cells in immunoregulation may be envisioned in this setting^54^. Additionally, while suppression of GVHD is a primary role for iNKT cells after postnatal transplantation, the virtual absence of this problem following prenatal transplantation of fetal liver hematopoietic cells may preclude the requirement for iNKT cell involvement in the current model. Future work will explore whether induced inflammation following IUHCT alters the contribution of iNKT cells to outcomes of prenatal transplantation.

In summary, this study demonstrates that iNKT cells are developmentally responsive to prenatally introduced allogeneic cells introduced during gestation leading to changes in allospecific Ly49 receptor expression and lineage diversity without alteration in iNKT cell responsiveness. Lineage-fate decisions in B6 iNKT cells appear to be intrinsically regulated and are not influenced by the extrinsic Balb/c environment while Balb/c cells are exquisitely sensitive to signals derived from B6 hematopoietic cells. Ultimately, these changes do not appear to be essential for graft tolerance as the absence of mature iNKT cells was compatible with sustained engraftment. Collectively, these findings lead to the conclusion that strain-specific cell-extrinsic signals dictate patterns of Ly49 receptor expression and lineage diversity in developing iNKT cells. Future studies will explore the nature of the dominant cell-extrinsic B6 signals influencing Balb/c iNKT cell lineage fates.

## Methods

### Animals

C57Bl/6J (B6) and B6.CD1d^−/−^ mice were purchased from Jackson laboratories (Bar Harbor, ME) while Balb/c mice were purchased from Charles River Laboratories (Wilmington, MA) and then maintained as a breeding colony at Cincinnati Children’s Hospital Medical Center.

### In utero transplantation model

Transplantation was performed as previously described^35^. Briefly, donor fetal liver cells were harvested from pregnant dams at E14 (embryonic day 14, day of plug = day 0). Using isoflurane anesthesia, the uterus was exposed following a midline laparotomy. Fetal livers were dissected and a single-cell suspension in phosphate buffered saline (PBS) was generated. Low-density mononuclear cells (LDMCs) were isolated using Ficoll density separation (Histopaque 1077, Sigma-Aldrich). Cells were washed with PBS and counted using trypan blue exclusion. A midline laparotomy was performed on recipient dams and a single-cell suspension of LDMCs in 5 μL PBS was injected intrahepatically into each E14 recipient fetus using a 100 μm beveled glass micropipette through the intact uterus. The abdomen was closed and pregnant dams housed individually.

### Evaluation of chimerism

Recipient fetuses were tracked for chimerism in peripheral blood (PB) serially starting at three weeks of age. LDMCs were isolated as described above. Antibodies used for determining chimerism were: CD45 (30-F11, eBioscience), H-2 Kd (SF1-1.1, BD Pharmingen) or H-2Dd (34-2-12), and H-2Kb (AF6-88.5, eBioscience). Dead cells were excluded using Hoechst 33528 (Invitrogen). Percentage non-erythroid donor chimerism was determined by the percentage of total CD45+ events that express donor MHC class I (H-2 Kd or H-2Dd in Balb/c → B6 or Balb/c → B6.CD1d^−/−^ chimeras, H-2Kb in B6 → Balb/c chimeras).

### Flow cytometry

LDMCs were stained with CD1d-PBS57 tetramer (NIH Tetramer Facility) for 1 hour, 4 °C along with identifying markers and analyzed by flow cytometry on a LSRII (BD Pharmingen). All antibodies were from eBioscience or BioLegend unless otherwise noted. CD3 (145-2C11), CD4(GK1.5), CD8(53.6.7), CD19(1D3), H-2Kd,(SF1-1.1), H-2Dd(34-2-12), H-2Kb(AF6-88.5), Ly49A(YE1), Ly49C/I(SW5E6), Ly49D (4E5), Ly49F(HBF-719), Ly49G(CWY-3), NK1.1 (PK136), TCR-β (H57-597), TCRvβ5.1/5.2(MR9-4), TCRvβ11(RR3-15), TCRvβ12(MR11-1).

For lineage analysis, surface staining was performed as indicated above followed by fixation and permeabilization according to foxp3 fix/perm kit instructions (eBioscience). Cells were blocked with 10% mouse serum in perm buffer prior to intranuclear staining for PLZF (Mags.21F7) and Tbet (eBio4B10).

Acid stripping was performed as previously described^28^. Briefly, after separation by ficoll as described above, cells were incubated for 1 minute with citrate buffer (0.133 M citric acid and 0.66 M Na_2_HPO_4_, pH 3.3). Treatment was stopped with an excess of RPMI media containing 10% FCS. Cells were washed three times with saline before staining as described above.

### KRN7000 Stimulation

KRN7000 (Enzo Life Sciences) was prepared and stored as described^55^. Animals were injected intravenously with 5 μg KRN7000 and organs were isolated 2 hours later. Surface staining was performed as described above. Cells were fixed and permeabilized following kit instructions (Cytofix/Cytoperm Kit, BD Pharmingen), then stained intracellularly for IFN-γ (XMG1.2) and IL-4 (11B11). iNKT cells were identified by expression of TCR-β and NK1.1 due to down-regulation of TCR expression.

### Statistics

Graphs were prepared using GraphPad Prism (San Diego, CA) with data represented as the mean of each group plus or minus standard deviation. Statistical comparisons were performed using a 2-tailed student *t* test assuming unequal variances, using Microsoft Excel software.

### Study approval

All experimental protocols and procedures were reviewed, approved, and carried out in accordance with guidelines set by the Institutional Animal Care and Use Committee at the Cincinnati Children’s Hospital Medical Center and followed National Institutes of Health regulations for laboratory animal use.

## Additional Information

**How to cite this article**: Strong, B. S. I. *et al.* Extrinsic allospecific signals of hematopoietic origin dictate iNKT cell lineage-fate decisions during development. *Sci. Rep.* **6**, 28837; doi: 10.1038/srep28837 (2016).

## Supplementary Material

Supplementary Information
