# Supplementary material for: Extrinsic allospecific signals of hematopoietic origin dictate iNKT cell lineage-fate decisions during development
Source: Sci Rep. 2016 Jun 29;6:28837. doi: 10.1038/srep28837 (PMC4926280; doi:10.1038/srep28837)
Supplement: Supplementary Information [file srep28837-s1.pdf]

Extrinsic allospecific signals of hematopoietic origin dictate iNKT cell lineage-fate decisions during development.

Beverly SI Strong, Tess J Newkold, Amanda E Lee, Lucas E Turner, Amir M Alhajjat, Jonathan W Heusel, Aimen F Shaaban

Supplemental Figure 1

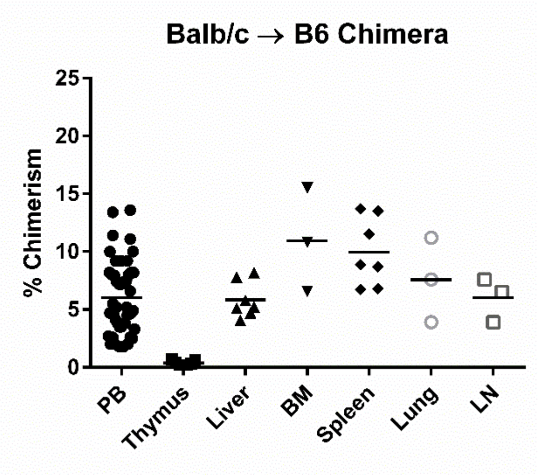

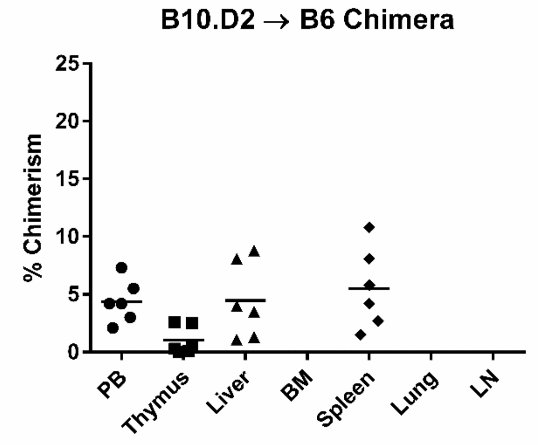

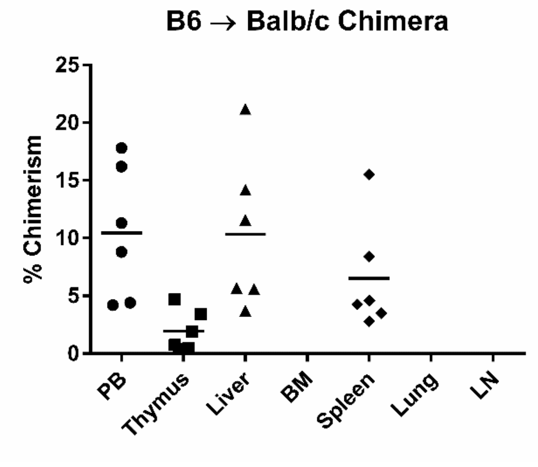

Supplemental Figure 2

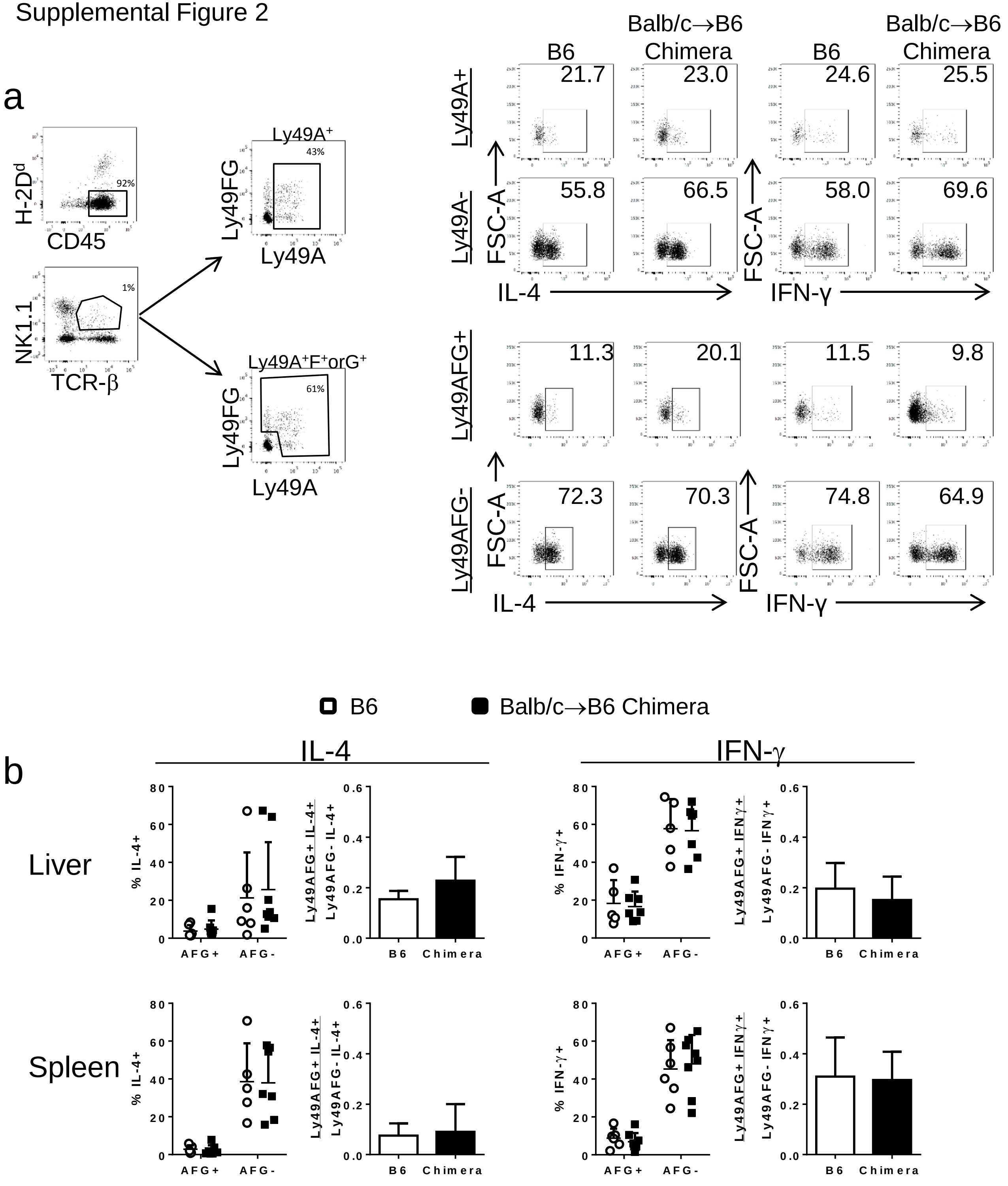

Supplemental Figure 3

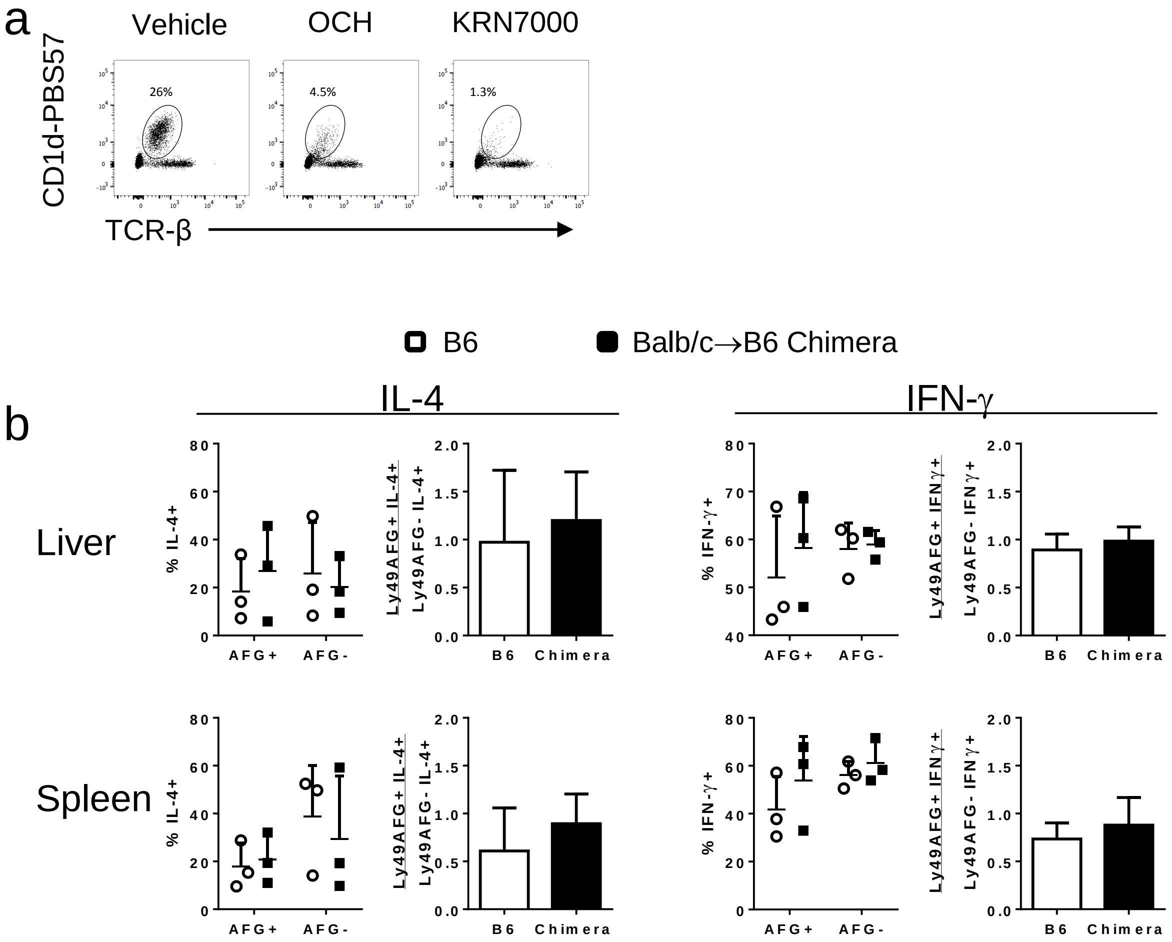

Supplemental Figure 1. Chimerism levels in all tissues tested. Chimerism levels for each tissue tested. Chimerism calculated as percent of CD45+ events expressing donor MHC class I (H-2K^d for Balb/c$\rightarrow$B6, H-2K^d for B10.D2 $\rightarrow$ B6, H-2K^b for B6 $\rightarrow$ Balb/c). Each data point is representative of one animal.

Supplemental Figure 2. Functional responsiveness of iNKT cells in prenatal chimeras. B6 control and Balb/c→B6 chimeras were injected intravenously with KRN7000 and NKT cells were analyzed two hours later for intracellular cytokine production. (A) iNKT cells were identified by co-expression of TCR-β and NK1.1 rather than CD1d-PBS57 tetramer due to down-regulation of TCR following stimulation. Representative dot plots showing IFN-γand IL-4 production by Ly49A+ vs Ly49A- or Ly49AFG+ vs Ly49AFG- iNKT cells. (B) Absolute and relative frequency of IFN-γ+ and IL-4+ iNKT cells are shown in graphs. Relative frequency of cytokine expression was calculated for donor reactive versus donor irrelevant phenotypes (Ly49AFG+ % Cytokine+/Ly49AFG- % Cytokine+). Each data point represents one animal.

Supplemental Figure 3. Functional responsiveness of iNKT cells in response to OCH glycolipid in prenatal chimeras. B6 control and Balb/c→B6 chimeras were injected intravenously with OCH and iNKT cells were analyzed two hours later for intracellular cytokine production. (A) iNKT cells were identified by co-expression of TCR-β and CD1d-PBS57 tetramer. Representative dot plots the ability to gate on tetramer positive cells following OCH but not KRN7000 stimulation. (B) Absolute and relative frequency of IFN-γ+ and IL-4+ iNKT cells are shown in graphs. Relative frequency of cytokine expression was calculated for donor reactive versus donor irrelevant phenotypes (Ly49AFG+ % Cytokine+/Ly49AFG- % Cytokine+). Each data point represents one animal.
